# Prevalence, Drug Resistance Patterns, and Associated Factors of Extended‐Spectrum Beta‐Lactamase–Producing Enterobacterales Among Food Handlers in Student Cafeterias at Arba Minch University, Southern Ethiopia

**DOI:** 10.1155/cjid/1139443

**Published:** 2026-07-10

**Authors:** Yigrem Alebachew, Zerihun Zerdo, Belayneh Regasa Dadi

**Affiliations:** ^1^ Department of Medical Microbiology, Arba Minch University, Arba Minch, Ethiopia, amu.edu.et

**Keywords:** Enterobacterales, Ethiopia, extended-spectrum *β*-lactamase, fecal carriage, food handlers, multidrug resistance

## Abstract

**Background:**

The emergence and spread of antimicrobial‐resistant Enterobacterales, particularly extended‐spectrum beta‐lactamase–producing Enterobacterales (ESBL‐PE), represent the major global public health concern. The objective of this study was to determine the fecal carriage, antimicrobial resistance pattern, and associated factors of ESBL‐PE among apparently healthy food handlers who work in the students’ cafeteria at Arba Minch University, Ethiopia.

**Method:**

An institution‐based cross‐sectional study was conducted from May 1 to August 30, 2025. A pretested, semistructured questionnaire was used to collect sociodemographic, clinical, behavioral, and other related data. Stool samples were collected from participants and inoculated on MacConkey agar. Bacterial colonies on the MacConkey agar were identified using standard biochemical tests. Screening and confirmation of extended‐spectrum *β-*lactamase production was done by Kirby Bauer’s disk diffusion method on Mueller–Hinton agar. The Statistical Package for the Social Sciences (SPSS) Version 25 was used to analyze the data. Bivariate and multivariate binary logistic regression analyses were used to assess the presence of association, and then the strength of the association was determined by using AOR and 95% confidence intervals, and *p* values at < 0.05 were considered statistically significant.

**Results:**

Overall prevalence of fecal carriage of ESBL‐PE was 51/319 (16%; CI 12.2–20.1). The most predominant ESBL‐PE was *Escherichia coli* (34/51, 66.7%) and *Klebsiella pneumonia* (14/51, 27.5%), followed by *Citrobacter freundii* (2/51, 3.9%) and *Proteus mirabilis* (1/51, 1.9%). All ESBL‐PE isolates in the present study were found to be multidrug‐resistant (MDR). High‐level resistance among ESBL‐producing Enterobacterales was observed against trimethoprim–sulfamethoxazole (88.2%), whereas meropenem remained highly effective with susceptibility ranging from 76.5% to 100% across different genera. Antibiotic use in the last three months (AOR = 2.485; CI: 1.12–5.51), lack of knowledge on the principle of safe food handling practice (AOR = 0.325; CI: 0.128–0.828), consumption of raw meat (AOR = 2.706; CI: 1.22–5.99), and drinking unpasteurized milk (AOR = 2.014; CI: 1.03–3.96) were significantly associated with fecal carriage of ESBL‐PE among food handlers.

**Conclusion:**

This study revealed a high prevalence of extended‐spectrum *β*‐lactamase–producing and MDR Enterobacterales among food handlers. Antibiotic use in the last three months, lack of knowledge of the principle of safe food handling practices, consumption of raw meat, and drinking unpasteurized milk were significantly associated with fecal carriage of ESBL‐PE among food handlers. Therefore, routine screening, health education, and strict infection prevention and control measures are essential to prevent the spread of ESBL‐PE and MDR‐resistant Enterobacterales from food handlers to the student community.

## 1. Background

Over the past decade, there have been an increasing number of reports on the fecal carriage of extended‐spectrum *β-*lactamase–producing Enterobacterales (ESBL‐PE) worldwide [1]. The highest ESBL‐PE carriage rate has been described in Asia [2]. In contrast, prevalence rates are lower in Europe and North America [1]. However, data on ESBL‐PE fecal carriage in sub‐Saharan Africa are limited, and a few available studies reported a prevalence ranging from 0.6 to 77.6% [3]. The World Health Organization (WHO) has identified ESBL‐PE as a pathogen of urgent importance for the development of new medications [4]. In order to prevent the emergence and spread of drug‐resistant bacteria, several control strategies have been proposed and implemented by WHO, but the incidence has still not been reduced [5, 6].

Drug‐resistant Enterobacterales primarily colonize the gut through contaminated food and water, and the rate of community colonization of drug‐resistant bacteria is, therefore, much higher and steadily increasing, even in low‐endemic settings [7]. Intestinal colonization with ESBL‐PE is an essential precursor for drug‐resistant infection and can contribute to unrecognized transmission in hospitals and communities [8]. Since ESBL‐PE colonization is endemic in most countries, clinicians have to take into account the risk of endogenous ESBL‐PE infections arising in the gut [9]. Gut colonization by ESBL‐PE may lead to subsequent endogenous infections, including sepsis, urinary tract infections, and deep abdominal infections [10]. This can lead to life‐threatening nosocomial and community‐acquired infections [11].

Food handlers in food‐serving facilities with poor personal hygiene can be infected/colonized by different enteropathogens who can then transmit to the public and environment [12]. Food handlers working in larger food venues are more epidemiologically significant, as they can spread numerous pathogens from their hands, hair, skin, digestive tracts, and respiratory tracts, as well as from contaminated food that they prepare and serve [13]. Quite often, they transmit pathogens, including drug‐resistant bacteria, to vulnerable or susceptible consumers, as they may carry these microbes as their skin flora or may be present in their nasal secretions or stool [14]. According to the CDC report, food handlers are responsible for about 20% of food‐related illnesses [15]. Anecdotally, this problem could be more in developing countries like Ethiopia, where supervision and monitoring of sanitary handling practices, good personal hygiene, regular medical check‐ups and decolonization procedures, and food safety regulations are not enforced.

The prevalence of ESBL‐PE among apparently healthy food handlers varies in different geographical areas, such as 4.8% in Japan [16], 13.2% in Kuwait [17], 9% in Qatar [18], 19.63% in Tunisia [19], 5% in Gambia [20], 21.7% in Gondar, Ethiopia [21], and 25% in Dilla, Ethiopia [22]. Previous studies have reported lack of training, antibiotic usage, compliance with completing prescribed antibiotics, and episodes of diarrhea as factors associated with fecal carriage of ESBL‐PE among food handlers [16–18, 21, 22]. Hitherto, there is no surveillance system available to screen fecal carriage of ESBL‐PE among food handlers within the study area. Therefore, it is imperative to generate local data on the prevalence, drug resistance patterns, and associated factors for WHO‐prioritized pathogens like ESBL‐PE to aid the development of prevention and control measures.

## 2. Methods and Materials

### 2.1. Study Design, Period, and Setting

An institution‐based cross‐sectional study was conducted from May 1 to August 30, 2025, in five student cafeterias at Arba Minch University, which is located 455 km south of Addis Ababa, the capital city of Ethiopia. Arba Minch University has six campuses, namely the Institute of Water Technology, College of Medicine and Health Sciences, College of Natural and Computational Sciences, College of Social Science and Humanities, and College of Agricultural Sciences. The multidisciplinary campuses have an enrollment capacity of more than 20 thousand students. Arba Minch University has six student cafeterias (one cafeteria per campus). According to information obtained from the human resources department, 553 food handlers are serving the cafeterias.

### 2.2. Eligibility Criteria

Inclusion criteria

• All food handlers who have worked at least one month in the cafeteria, irrespective of sex and age.
• Those who were willing to participate in the study during the study period.

Exclusion criteria

• Food handlers who took any antibiotics prior to two weeks of commencement of the study.
• Those who were unable to give stool samples.

### 2.3. Sample Size Determination and Sampling Technique

The sample size was calculated by using the single population proportion formula. Using 25% prevalence obtained from the previous study conducted in Dilla, Ethiopia [22], the total study sample size was calculated as 319 study subjects.

### 2.4. Sampling Technique

A simple random sampling technique was employed to recruit the subjects (roster lists of food handlers were obtained from the Human Resource Department of Arba Minch University). The study participants were selected from the sampling frame after giving a unique identification number. Every person was chosen randomly until the final sample size was reached.

### 2.5. Data Collection and Laboratory Processing

A semistructured and pretested questionnaire was developed based on related published studies, with certain modifications. The questionnaire was initially prepared in English, translated into Amharic, and then back‐translated into English to ensure the accuracy of the translation. Data collection was carried out by trained data collectors through face‐to‐face interviews under the supervision of the principal investigator.

### 2.6. Sample Collection and Transportation

The study participants were instructed to collect approximately 2 grams of stool into a clean and leak‐proof container [21]. Following collection, all stool specimens were labeled with the sample number, time, and date of sample collection [21]. The specimens were then immediately transported to the Medical Microbiology and Parasitology Laboratory, Arba Minch University, using Cary–Blair transport medium within 1 h of collection.

### 2.7. Bacterial Isolation

A loopful of stool sample was inoculated onto MacConkey agar and incubated at 37°C for 18–24 h. After incubation, isolates were identified phenotypically based on colony characteristics (shape, margin, elevation, consistency, and color), motility, Gram staining (HiMedia Laboratories Pvt. Ltd., Mumbai, India), and a series of standard biochemical tests (61). Biochemical tests including indole, citrate, urease, oxidase tests, triple sugar iron (TSI), and hydrogen sulfide (H_2_S) production were used to identify members of the Enterobacterales family [21].

### 2.8. Antimicrobial Susceptibility Testing

The antimicrobial susceptibility test was conducted using the Kirby–Bauer disk diffusion technique in accordance with the Clinical and Laboratory Standards Institute (CLSI) 2021 guidelines. To prepare the inoculum, portions of similar test organisms were selected and suspended in sterile normal saline using a sterile wire loop. The density of the suspension was adjusted by comparing it to a 0.5 McFarland barium sulfate standard. The surface of Mueller–Hinton agar (Oxoid, UK) was uniformly seeded with the test organisms. The plates were then incubated at 37°C for 24 to 48 h to allow the antibiotic to diffuse from the impregnated paper discs, creating a concentration gradient. The diameters of the zones of inhibition surrounding the discs were measured to the nearest millimeter using a ruler and classified as sensitive, intermediate, or resistant according to CLSI 2021 standards. The antimicrobial susceptibility test included the following antibiotic discs: gentamicin (GEN; 10 μg), ciprofloxacin (CIP; 30 μg), chloramphenicol (CHL; 30 μg), and trimethoprim/sulfamethoxazole (23.75/1.25 μg) (Oxoid, UK) [23].

### 2.9. Screening of ESβL‐Producing Enterobacterales

The susceptibility of Gram‐negative bacteria was tested against third‐generation cephalosporins, including ceftriaxone, cefotaxime, and ceftazidime, using the routine Kirby–Bauer disk diffusion method on Mueller–Hinton agar. Isolates that showed zones of inhibition of < 25 mm for ceftriaxone (30 μg), < 27 mm for cefotaxime (30 μg), and < 22 mm for ceftazidime (30 μg) (for the three or one of the antibiotic discs) were considered positive for extended‐spectrum beta‐lactamase (ESBL) production [23].

### 2.10. Double‐Disk Synergy Test (DDST)

A disc of amoxicillin–clavulanate (30 μg) along with third‐generation cephalosporins, including ceftriaxone (30 μg), cefotaxime (30 μg), and ceftazidime (30 μg), was used for confirmation. The amoxicillin–clavulanate disc was placed at the center of the inoculated Mueller–Hinton agar plate, and discs of ceftriaxone, cefotaxime, and ceftazidime were placed 20–30 mm away from the central disc. An enhanced zone of inhibition toward the amoxicillin–clavulanate disc was considered characteristic of a potential ESBL‐producing isolate [23].

### 2.11. Data Quality Assurance

Data quality was ensured throughout the entire process, from data collection to laboratory identification, by adhering to standard operating procedures. Prior to use, the expiration dates of antibiotic discs, reagents, and media were checked. Culture media were prepared according to the manufacturer’s instructions, and sterility was verified both by observing bacterial growth and by overnight incubation of 5% of the prepared media at 35°C–37°C. Any batches of media that exhibited growth were discarded and re‐prepared. After sterility testing, the culture media were inspected for cracks, thickness, impurities, freezing, and bubbles. As a quality control for ESBL‐producing Enterobacterales detection, ESBL‐positive *Klebsiella pneumoniae* ATCC 700603 and ESBL‐negative *Escherichia coli* ATCC 25922 control strains were used.

Data collectors received training from the investigators prior to data collection. A pretest, comprising 5% of the sample size, was conducted on food handlers working in the student cafeteria at Wolayita Sodo University one week before the official data collection period. To ensure the consistency of the questionnaire, the form was translated into Amharic and then back‐translated into English. Stool specimens were collected and transported using Cary–Blair medium as soon as possible after collection, and any specimens were stored in the refrigerator if processing was delayed.

### 2.12. Statistical Analysis

Data analysis was performed using IBM SPSS Statistics for Windows, Version 25 (IBM Corp., Armonk, NY, USA). The dependent variable was the prevalence of fecal carriage of ESBL‐PE. Sociodemographic and related factors were initially summarized using descriptive statistics. Inferential statistical analysis was conducted using bivariable and multivariable binary logistic regression. In the first step, the data were subjected to bivariable analysis, and only covariates with a *p* value ≤ 0.25 were included in the multivariable analysis. The strength of association was determined using adjusted odds ratios (AORs) with 95% confidence intervals (CIs), and statistical significance was set at *p* ≤ 0.05. The results were presented using tables, graphs, and text.

## 3. Results

### 3.1. Sociodemographic Characteristics of the Study Participants

A total of 319 food handlers were included in this study. Of these, the majority of the study participants were female (293, 91.8%). More than half of the participants (163, 51.1%) were aged between 31 and 40 years, with a mean age of 36.84 years and a standard deviation of ± 7.04 years. The educational status of more than half (186, 58.3%) of the respondents had completed a college diploma (see Table 1).

**TABLE 1:** Sociodemographic characteristics of food handlers at Arba Minch University, southern Ethiopia.

Variables	Categories	Frequency	Percentage
Sex	Male	26	8.2
	Female	293	91.8

Age	21–30	69	21.6
	31–40	163	51.1
	> 40	87	27.3

Marital status	Married	280	87.8
	Singled	22	6.9
	Divorced	10	3.1
	Windowed	7	2.2

Educational status	No formal education	10	3.1
	Primary school	36	11.3
	Secondary school	87	27.3
	College diploma and above	186	58.3

Family size	1–5	293	91.8
	> 5	26	8.2

Family income	4000–6699	92	28.8
	7000–9999	180	56.4
	10,000–13,000	47	14.7

### 3.2. Work‐Related Factors, Hygiene, Dietary, and Environmental Factors

Most participants had more than five years of service (256, 80.3%). Nearly two‐thirds of the respondents, 198 (62.1%), reported receiving training on food handling practices, and 173 (54.2%) had knowledge of safe food handling principles. Regarding personal hygiene, 265 (83.1%) of respondents had trimmed fingernails. All respondents used tap water for drinking and had access to toilets. Hand washing after toilet use, after touching dirty material, and before touching food was practiced by all participants, with 230 (72.1%) washing hands with soap and water after using the toilet and 170 (53.3%) washing hands with soap and water before touching food. However, only 147 (46.1%) of participants reported washing their hands with soap and water after touching dirty materials. Regarding dietary and environmental exposure, 96 (30.1%) of respondents reported drinking unpasteurized milk, 193 (60.5%) consumed raw meat, and 150 (47%) ate raw vegetables. Nearly half of the participants, 158 (49.5%), consumed food from street vendors. Additionally, 121 (37.9%) of respondents reported having pet animals at home (Table 2).

**TABLE 2:** Work‐related, hygiene, dietary, and environmental factors for fecal carriage of ESBL‐PE among food handlers at Arba Minch University, southern Ethiopia.

Variables	Categories	Frequency	Percentage
Service year	1–5 year	63	19.7
	> 5 year	256	80.3

Training on food handling practice	Yes	198	62.1
	No	121	37.9

Know principle of safe food handling practice	Yes	173	54.2
	No	146	45.8

Finger nail status	Trimmed	265	83.1
	Non trimmed	54	16.9

Drink unpasteurized milk	Yes	96	30.1
	No	223	69.9

Consumed raw meat	Yes	193	60.5
	No	126	39.5

Ate raw vegetables	Yes	150	47
	No	169	53

Consumed street vendor food	Yes	158	49.5
	No	161	50.5

Presence of pet animal	Yes	121	37.9
	No	198	62.1

### 3.3. Clinical Factors

Concerning health‐related characteristics, 190 (59.6%) of participants had undergone medical check‐ups every six months. A small proportion, 10 (3.1%), reported a history of hospital admission in the last three months. Antibiotic use in the last three months was reported by 96 (30.1%) of respondents. A history of urinary tract infection was reported by 87 (27.3%) of participants, while 46 (14.4%) reported experiencing diarrhea in the last three months (Table 3).

**TABLE 3:** Clinical factors for fecal carriage of ESBL‐PE among food handlers at Arba Minch University, southern Ethiopia.

Variables	Categories	Frequency	Percentage
Medical check‐up every six months	Yes	190	59.6
	No	129	40.4

History of hospital admission in the last three months	Yes	10	3.1
	No	309	96.9

History of antibiotic use in the last three months	Yes	96	30.1
	No	223	69.9

History of urinary tract infection in the last three months	Yes	87	27.3
	No	232	72.7

History of diarrhea in the last three months	Yes	46	14.4
	No	273	85.6

### 3.4. Prevalence of Enterobacterales Isolates From the Stool

All of the study participants had culture‐positive results yielding a total of 336 bacterial isolates. Of these results, 302 participants contained a single bacterial isolate, while 17 participants carried two bacterial isolates. *E. coli* was the most prevalent isolate 202/336 (60.1%), followed by *K. pneumoniae* 110/336 (32.7%), *Citrobacter freundii* (9, 2.7%), *Enterobacter cloacae* (7, 2.1%), *Proteus mirabilis* (5, 1.5%), and *Morganella morganii* (3, 0.9%) (Table 4).

**TABLE 4:** Proportion of Enterobacterales isolates from food handlers in student cafeterias at Arba Minch University, southern Ethiopia.

Bacterial isolates	Frequency	Percentage
*E. coli*	202	60.1
*K. pneumoniae*	110	32.7
*C. freundii*	9	2.7
*E. cloacae*	7	2.1
*P. mirabilis*	5	1.5
*M. morganii*	3	0.9
Total	336	100

### 3.5. Fecal Carriage of ESBL‐Producing Enterobacterales Among Food Handlers

In this study the overall fecal carriage of ESBL‐PE among food handlers was 51/319 (16%). Among the 51 ESBL‐PE isolates recovered, *E. coli* accounted for 34/51 (66.7%), followed by *K. pneumoniae* 14/51 (27.5%), *C. freundii* (2/51, 3.9%), and *P*. *mirabilis* (1/51, 1.9%) (Table 5).

**TABLE 5:** Distribution of ESBL‐PE among food handlers in student cafeterias at Arba Minch University, southern Ethiopia.

Bacterial isolates	Frequency	Percentage
*E. coli*	34	66.7
*K. pneumoniae*	14	27.5
*C. freundii*	2	3.9
*P. mirabilis*	1	1.9
Total	51	100

### 3.6. Antimicrobial Resistance Pattern of ESBL‐PE

In this study, a high level of resistance to trimethoprim–sulfamethoxazole was observed among ESBL‐PE. Most of ESBL‐PE was highly susceptible to meropenem (76.5%–100%). However, *E. coli* and *K. pneumoniae* were resistant to meropenem, with resistance rate of 23.5% and 14.3%, respectively (Table 6).

**TABLE 6:** Antimicrobial resistance pattern of ESBL‐PE among food handlers in student cafeterias at Arba Minch University, southern Ethiopia.

Antibiotics	*E. coli* (*n* = 34)		*K. pneumoniae* (*n* = 14)		*C. freundii* (*n* = 2)		*P. mirabilis* (*n* = 1)	
	*S* (%)	*R* (%)	*S* (%)	*R* (%)	*S* (%)	*R* (%)	*S* (%)	*R* (%)
AUG	22 (64.7%)	12 (35.3%)	9 (64.3%)	5 (35.7%)	1 (50%)	1 (50%)	0	1
CIP	23 (67.6)	11 (32.4%)	10 (78.6%)	4 (28.6%)	0 (0)	2 (100%)	1	0
CHL	17 (50%)	17 (50%)	5 (35.7%)	9 (64.3%)	0 (0)	2 (100%)	1	0
GEN	23 (67.6%)	11 (32.4)	10 (71.4%)	4 (28.6%)	0 (0)	2 (100%)	1	0
CRO	0 (0)	34 (100%)	0 (0)	14 (100%)	0 (0)	2 (100%)	0	1
CAZ	0 (0)	34 (100%)	0 (0)	14 (100%)	0 (0)	2 (100%)	0	1
STX	4 (11.8%)	30 (88.2%)	1 (7.1%)	13 (92.9%)	0 (0)	2 (100%)	0	1
CTX	0 (0)	34 (100%)	0 (0)	14 (100%)	0 (0)	2 (100%)	0	1
MER	26 (76.5%)	8 (23.5%)	12 (85.7%)	2 (14.3%)	2 (100%)	0 (0)	1	0

*Note: S* = sensitive, *R* = resistant, AUG = Augmentin (amoxicillin–clavulanate), CIP = ciprofloxacin, GEN = gentamicin, CRO = ceftriaxone, CTX = cefotaxime, CAZ = ceftazidime, STX = trimethoprim–sulfamethoxazole, MER = meropenem, CHL = chloramphenicol.

### 3.7. Multidrug‐Resistance (MDR) Pattern of Enterobacterales Isolates Among Food Handlers

In this study high MDR *E. coli* was found, followed by *K. pneumoniae*. A low number of MDRs were observed in *C. freundii* and *Proteus* ssp. (see Table 7).

**TABLE 7:** Multidrug‐resistance profile of Enterobacterales isolates among food handlers at Arba Minch University in student cafeterias, southern Ethiopia.

Isolate *N* = 51	Level of resistance							
	R0	R1	R2	R3	R4	R5	R6	MDR ≥ R3
*E. coli* (*N *= 34)	0	0	0	23	4	5	2	34
*K. pneumoniae* (*N *= 14)	0	0	0	9	3	1	1	14
*C. freundii* (*N *= 2)	0	0	0	0	0	2	0	2
*P. mirabilis* (*N* = 1)	0	0	0	1	0	0	0	1
Total (*N* = 51)	0	0	0	33	7	8	3	51

*Note:* R0 = sensitive to all classes of antibiotics, R1 = resistance to one class, R2 = resistance to two classes, R3 = resistance to three classes, R4 = resistance to four classes, R5 = resistance to five classes, R6 = resistance to six classes, MDR ≥ 3 = resistance to three or more classes of antibiotics.

### 3.8. Factor Associated With Fecal Carriage of ESBL‐PE Among Food Handlers

Antibiotic use in the last three months (AOR = 2.485; CI: 1.12–5.51), lack of knowledge on the principle of safe food handling practice (AOR = 0.325; CI: 0.128–0.828), consumption of raw meat (AOR = 2.706; CI: 1.22–5.99), and drinking unpasteurized milk (AOR = 2.014; CI: 1.03–3.96) were factors associated with fecal carriage of ESBL‐PE among food handlers (Table 8).

**TABLE 8:** Factors associated with fecal carriage ESBL‐PE among food handlers at Arba Minch University in student cafeterias, southern Ethiopia.

Variables	Categories	Number of tested (*N* = 319)	ESBL positive	ESBL negative	Bivariate analysis		Multivariate analysis	
					COR (95%) CI	*p* value	AOR (95%) CI	*p* value
Sex	Male	26	4	22	0.9 (0.3–2.9)	0.930		
	Female	293	47	246	1			

Age	21–30	69	10	59	1			
	31–40	163	26	137	1.1 (0.5–2.5)	0.779		
	> 40	87	15	72	1.1 (0.5–2.9)	0.642		

Marital status	Single	22	3	19	1			
	Married	278	44	234	1.2 (0.3–4.2)	0.768		
	Divorced	10	2	8	1.6 (0.2–11.4)	0.648		
	Windowed	9	2	7	1.8 (0.2–13.2)	0.59		

Educational status	No formal education	10	1	9	0.5 (0.07–4.4)	0.55		
	Elementary education	36	5	31	0.8 (0.3–2.1)	0.626		
	High school	87	13	74	0.8 (0.4–1.7)	0.639		
	Diploma and above	186	32	154	1			

Family size	1–5 years	293	49	244	2.3 (0.5–10)	0.269		
	> 5 years	26	2	24	1			

Average monthly income	4000–6999	92	26	76	0.9 (0.4–2.2)	0.799		
	7000–9999	180	16	154	0.7 (0.3–1.6)	0.42		
	10,000–13,000	47	9	38	1	1		

Service year	1–5	63	11	52	1.1 (0.5–2.4)	0.722		
	> 5	256	40	216	1			

Training on safe food handling practice	Yes	198	22	176	1		1	
	No	121	29	92	2.5 (1.4–4.6)	0.03^∗^	0.716 (0.296–1.730)	0.458

Knowing the principle of safe food handling practice	Yes	173	15	158	1		1	
	No	146	36	110	3.4 (1.8–6.0)	0.00^∗^	0.325 (0.128–0.828)	0.019^∗^

Medical check‐up	Yes	190	26	164	1			
	No	129	25	104	1.5 (0.4–2.8)	0.175^∗^	0.601 (0.301–1.199)	0.149

History of hospital admission	Yes	10	1	9	0.5 (0.07–4.6)	0.604		
	No	309	50	259	1			

History of urinary tract infection	Yes	87	22	65	2.4 (1,2–4.4)	0.006^∗^	1.353 (0.592–3.092)	0.474
	No	232	29	203	1			

History of antibiotic use in the last three months	Yes	96	28	68	3.58 (1.9–6.6)	0.00^∗^	2.485 (1.122–5.505)	0.025^∗^
	No	223	23	200	1			

History of diarrhea in the last three months	Yes	46	13	33	2.4 (1.2–5.0)	0.016^∗^	1.611 (0.702–3.698)	0.261
	No	273	38	235	1			

Finger nail status	Trimmed	265	40	225	1			
	Non‐trimmed	54	11	43	0.69 (0.31–1.5)	0.337		

Drink unpasteurized milk	Yes	96	25	71	2.7 (1.4–4.9)	0.002^∗^	2.014 (1.025–3.955)	0.042^∗^
	No	223	26	197	1			

Consume raw meat	Yes	193	41	152	3.1 (1.5–6.5)	0.002^∗^	2.706 (1.22–5.988	0.014^∗^
	No	126	10	116	1		1	

Eaten raw vegetables	Yes	154	34	120	1.8 (0.9–3.2)	0.068^∗^	1.721 (0.865–3.422)	0.122
	No	165	17	148	1		1	

Consume street vendor food	Yes	158	27	131	1.2 (0.6–2.1	0.595		
	No	161	24	137	1			

Presence of pet animals	Yes	121	20	101	1.1 (0.6–1.9)	0.837		
	No	198	31	167	1			

^∗^Indicate statistically significant at *p* < 0.25 in bivariate and at < 0.05 in multivariate logistic regression analysis.

## 4. Discussion

In this study, the prevalence of fecal carriage of ESBL‐PE among food handlers was found to be 16.0% (CI: 12.2%–20.1%). This result aligns with studies done in *Argentina* [24], Tunisia [19], and Kuwait [17]. However, the prevalence noted in the present study is lower than the rates found in South India (33.8%) [25], Dilla, Ethiopia (25.3%) [22], and Gondar, Ethiopia (21.7%) [21]. This variation may be due to differences in research design, sample size, diagnostic techniques, or disparities in antibiotic usage and hygiene practices among the populations studied. Conversely, the prevalence of ESBL‐PE carriage reported in this study is greater than those reported in Gambia (5%) [20], Qatar (9%) [18], and Japan (4.8%) [16]. These discrepancies could be attributed to differences in methodologies, especially the laboratory techniques utilized for the confirmation of ESBL, along with geographical and socioeconomic factors that affect antimicrobial exposure, sanitation, and food safety standards.

In our study, isolates showed significant resistance levels to trimethoprim–sulfamethoxazole with a resistance rate of 88.2% in *E. coli* and 92.9% in *K. pneumoniae*. These results are similar to those reported from Gondar, Ethiopia [21], Gambia [20], Nigeria [26], Argentina [24], and Switzerland [27]. In our study, a minimal level of resistance to meropenem was noted, with rates ranging from 0% to 4.5%, which aligns with findings from studies in Gondar, Ethiopia [21], and Dilla, Ethiopia [22].

In the current study, the overall prevalence of MDR Enterobacterales was found to be 100%. This result is consistent with research conducted in Gondar, Ethiopia [21], and Chad [1]. However, the prevalence observed in this study is higher than reported from Gambia [20], Qatar [18], and Kenya [28]. These discrepancies may be due to inappropriate antibiotic usage, increased intake of animal products treated with antimicrobial agents, the growing prevalence of MDR strains over time, and differences in the sociodemographic and clinical profiles of the study populations.

All ESBL‐PE isolates were resistant to cefotaxime, ceftriaxone, and ceftazidime. This finding is consistent with studies conducted in Gambia [20], Gondar, Ethiopia [21], and Dilla, Ethiopia [22]. In the present study, ESBL‐PE isolates were not only completely resistant to third‐generation cephalosporin *β*‐lactam antibiotics but also exhibited high resistance to non–*β*‐lactam antibiotics, particularly trimethoprim–sulfamethoxazole, with a resistance rate of 87.1%. This observation is in agreement with findings reported from Gondar, Ethiopia [21], and Dilla, Ethiopia [22]. Meropenem was the most effective antimicrobial agent against ESBL‐PE isolates, demonstrating a susceptibility rate ranging from 75% to 100%. Despite this, all ESBL‐PE isolates in the present study were classified as MDR. The MDR nature of ESBL‐PE may be attributed to plasmid‐mediated resistance mechanisms, whereby ESBL genes are present with other multiple antimicrobial resistance determinants in the bacteria. These resistance genes can be readily disseminated among Enterobacterales through horizontal gene transfer mechanisms, including conjugation, transduction, and transformation.

Fecal carriage of ESBL‐PE was significantly higher among food handlers who reported antibiotic use in the last three months compared with those who had not used antibiotics during that period. This result aligns with findings from studies conducted in Gondar, Ethiopia [21], Dilla, Ethiopia [22], Gambia [7], Dutch [29], and China [30]. This association may be due to the selective pressure exerted by antibiotic exposure, which favors the survival and proliferation of resistant bacteria, thereby facilitating the persistence and spread of ESBL‐producing organisms within the community.

Lack of knowledge regarding safe food handling practices was independently associated with fecal carriage of ESBL‐PE in the present study. This finding is consistent with studies conducted in Gondar, Ethiopia [21], Gambia [7], and Ghana [31], where insufficient food safety knowledge was identified as a significant risk factor for fecal carriage of resistant Enterobacterales. Inadequate knowledge of food hygiene may increase the risk of fecal–oral transmission of resistant organisms through poor hand hygiene, cross‐contamination, and unsafe food preparation practices.

Consumption of raw meat was another significant factor associated with ESBL‐PE carriage. This finding is consistent with studies conducted in Gondar, Ethiopia [21], Dilla, Ethiopia [22], Italy [32], and Turkiye [33]. Raw or undercooked meat is a well‐recognized reservoir of ESBL‐PE, particularly in settings where antibiotic use in animal husbandry is poorly regulated. Consequently, consumption of raw meat may facilitate intestinal colonization with antimicrobial‐resistant bacteria. Similarly, drinking unpasteurized milk was significantly associated with fecal carriage of ESBL‐PE. Food handlers who consumed unpasteurized milk were about two times more likely to be colonized with ESBL‐producing organisms compared to those who did not. This finding is consistent with previous studies conducted in Gondar, Ethiopia [21], Dilla, Ethiopia [22], and Germany [34]. This may be due to unpasteurized milk serving as a vehicle for transmission of antimicrobial‐resistant bacteria due to contamination during milking, handling, or storage.

## 5. Conclusion

The most common Enterobacterales isolated from this study were *E. coli* and *K. pneumonia*, with a high prevalence of ESBL‐PE and MDR Enterobacterales among food handlers. Meropenem was the most effective antibiotic when compared to other tested antiobiotics. Factors such as antibiotic use in the last three months, lack of knowledge of the principle of safe food handling practices, consumption of raw meat, and drinking unpasteurized milk were significantly associated with fecal carriage of ESBL‐PE among food handlers in this study. Therefore, routine screening, health education, strict infection prevention, and control measures are essential to prevent the spread of ESBL‐PE and MDR‐resistant Enterobacterales from food handlers to the community.

## Author Contributions

Authors carried out proposal development, data collection, data analysis and drafted the manuscript.

## Funding

No funding was received for this manuscript.

## Disclosure

All authors read and approved the final manuscript. All authors agreed to publish the article.

## Ethics Statement

Ethical clearance was obtained from the Arba Minch University College of Medicine and Health Sciences Institutional Review Board. Permission was obtained from each Arba Minch University campus prior to the commencement of the study, and written informed consent was obtained from all participants. After being fully informed about the objectives and design of the study, potential participants were asked to complete a standard written consent form and sign a document indicating their voluntary participation without coercion. All results were kept confidential, and participants had the full right to refuse participation. Additionally, the participants’ specimens were analyzed solely for the purposes outlined in the study.

## Conflicts of Interest

The authors declare no conflicts of interest.

## Data Availability

Data are available on request due to privacy/ethical restrictions.
